# The Quality of Life and Its Relationship With Systemic Family Dynamics and Mental Health in Senior High School Students From Shaanxi, China

**DOI:** 10.3389/fpubh.2022.833561

**Published:** 2022-03-31

**Authors:** Zhe Yang, Yijiang Shang, Ying Liang, Haiyue Zhang, Yifan Yang, Yue Wang, Lei Shang, Yuhai Zhang

**Affiliations:** ^1^Department of Health Statistics and Ministry of Education Key Lab of Hazard Assessment and Control in Special Operational Environment, Fourth Military Medical University, Xi'an, China; ^2^Academy of Arts, Xi'an University of Architecture and Technology, Xi'an, China

**Keywords:** quality of life, systemic family dynamics, mental health, senior high school students, structural equation modeling

## Abstract

**Objective:**

To explore the quality of life (QoL) status of senior high school students in the Shaanxi Province and the relationship of the QoL with systemic family dynamics and mental health.

**Methods:**

This was a cross-sectional observational study in a sample of 1,402 senior high school students; students were asked to complete a questionnaire which comprised the 36-item Short Form Health Survey (SF-36) to assess the QoL, the Self-rating Scale of Systemic Family Dynamics (SSFD) for assessing family functioning, the Symptom Checklist-90-Revised (SCL-90-R) for assessing mental health and general demographic variables.

**Results:**

Grades of senior high school students were defined as Grade 1 (first year), Grade 2 (second year), and Grade 3 (third year). Compared to Grade 3 students, Grade 1 students had higher scores in physical functioning, role-emotional, bodily pain, and reported health transition of the QoL over the last 1 year (*P* < 0.05). Multiple linear regression showed that the place of residence, subscales of systemic family dynamics, somatization, and depression significantly affected the total QoL score. The results of SEM show that the SCL-90-R score fully mediated the association between the SSFD score and SF-36 score (indirect effect coefficient 0.055; 95% CI, 0.012–0.106; *P* = 0.042).

**Conclusion:**

The QoL score of senior high school students was low, particularly that of Grade 3 students, who consequently need more emotional support. By improving and enhancing systemic family dynamics, the QOL of high school students will improve and discovering and addressing their mental health problems will be easier.

## Introduction

Quality of life (QoL) is a global health indicator that provides information that is not typically reflected in the usually used clinical instruments; it provides information on the physical, psychological, and social subscales of people's life (1). Similarly, the pursuit of a better QoL is considered a fundamental mission of psychology (2). Students going to senior high schools are in the most important transitional stage of adolescence, and their QoL at this stage will directly or indirectly affect their adult life (3). The two most important factors affecting the QoL of senior high school students are family and mental health according to prior research (4).

Regarding family as an influencing factor of the QoL, some of the family factors include family structure, family atmosphere, and family members' psychological state and behavior, which reflect the interaction among and psychological process of family members (5, 6). Accumulating research has reported that adolescents' systemic family dynamics are closely related to their QoL and that there may be interaction and underlying causality between them (7–10). Numerous studies also show that the family togetherness and general health status were positively associated with better health-related QoL among Chinese senior high school students (11). Prior studies have revealed that good family atmosphere and family upbringing can maximize the QoL of students (12).

Regarding mental health as an influencing factor of the QoL, a study recommended that mental health promotion should be prioritized in improving the overall QoL of adolescents both in Japan and China (13). Stheneur et al. found that depression significantly influenced the QoL of adolescents (14). Currently, there are few researches on psychology and the QoL, and most of them focus on the QoL of patients with certain diseases, such as anxiety, back pain, obesity, and irritable bowel syndrome (15–18) among others. Since students in senior high school need to take a standardized entrance exam for admission in a good college or university, they are more prone to experiencing psychological, emotional, academic, and interpersonal pressure, which greatly affects their QoL (19, 20).

We assume that sociodemographic variables, mental health, and systemic family dynamics are related to QoL in senior high school students. Thus, in the present study, we aimed to assess the factors associated with family dynamics and mental health that affect the QoL (21) and the correlation among systemic family dynamics, mental health, and QoL in senior high school students.

## Methodology

### Study Design

We conducted a cross-sectional study that included a convenience sample of senior high school students (*n* = 1,402) from three senior high schools in the Shaanxi Province, China. All questionnaires were paper-based and self-administered. Data were collected between August 2014 and May 2015.

All research participants provided written informed consent and were informed of their right to withdraw anytime during the study. The study conforms with the local legislation and institutional requirements. Data obtained from all participants were kept confidential and anonymous to protect their privacy. Out of all students, 1,367 students completed the questionnaire, amounting to a response rate of 97.5%. Fifty percentage items in 11 questionnaires were not responded, which could be attributed to respondents' delay or discomfort in the process of answering the questionnaire; these complications were very individual-specific and were thus deleted. After excluding these inadequately responded questionnaires, 1,356 effective questionnaires were used for statistical analysis. Sixty-seven questionnaires had missing values. Missing data were imputed with multiple imputations.

### Survey Instruments

We used a questionnaire package to get data material, which included four parts: sociodemographic variables, the 36-item Short Form Health Survey (SF-36), the Self-rating Scale of Systemic Family Dynamics (SSFD), and the Symptom Checklist-90-Revised (SCL-90-R). A total of 1,402 high school students participated in this survey. The sociodemographic variables considered included gender, family composition, monthly income, place of residence, and each parent's education level and occupation.

The QoL was assessed using the Chinese version of the Medical Outcomes Study's SF-36 (22). This SF-36 comprised 36 items that measured the following eight health subscales: physical functioning, role-physical, bodily pain, general health perceptions, vitality, social functioning, role-emotional and mental health. Besides, reported health transition was used to measure the change in health status over 1 year. All these items comprehensively reflected the QoL of the respondents. The score of each subscale was converted to a standard score ranging from 0 to 100, with the highest score indicating the best QoL. The SF-36 reportedly has good reliability and validity among various Chinese students (23).

The SCL-90-R was used to assess the mental health status of the students. It is among the most widely used and well-known self-report instruments designed to measure psychological problems and symptoms of psychopathology; furthermore, it has been extensively used in Chinese studies (24). It comprises 90 items across nine subscales: somatization (12 items), obsessive-compulsive disorder (10 items), interpersonal sensitivity (9 items), depression (13 items), anxiety (10 items), hostility (6 items), phobic anxiety (7 items), paranoid ideation (6 items), psychoticism (10 items), and others (7 items). A Likert-type question–answer model is used in SCL-90-R (none = 0, too much = 4) (25). The nine scales of the SCL-90-R are also reliable in adolescents (26). The mean score of all items in each subscale represents the severity of that mental health issue in the person (27). Scores ≥2.5 for each subscale were defined as being suggestive of potential mental health problems (28).

The SSFD is the only localized family dynamics scale based on the Heidelberg family dynamics theory. It was compiled and revised in China (29–31). It is self-completed by the individual and thus represents their perception of the dynamics of their family. The revised scale has four subscales, which include 29 items reflecting the perceived dynamics of the family. The four subscales are family atmosphere (11 questions), personalization (8 questions), system logic (6 questions), and disease concepts (4 questions). The family atmosphere category covers the emotionality of communication within the family. Questions include the variables of expressing strong feelings, expressing care, and communication. The personalization category asks about whether the children had free development in terms of forming opinions and questions how free members feel regarding their interests, decisions, time spent, and personality. The system logic category asks about the extent to which the family uses “black and white” logic to distinguish items/issues. The questions in this category pertain to the extent to which items/issues are judged as right or wrong, good or bad, and very bad or very good. Finally, the disease concepts category asks about family's awareness of disease factors. The questions in this category pertain to SCL-90-R the occurrence of mental illness being related to the family environment, interpersonal relationships, self-adjustment, and personal lifestyle. Lower scores on the first three subscales reflect better family dynamics, as do higher scores on disease concepts.

In the SSFD, the scale has good cultural adaptability and reflects the family situation in China comprehensively and systematically (32). It has been widely used to assess the characteristics of family dynamics and the changes in family dynamics before and after controlling family influencing factors (33). The Likert-type question–answer model was used in the SSFD (completely inconsistent = 0, completely consistent = 4).

### Procedures

Before the formal survey, the class teacher or a counselor explained the significance and purpose of the survey to the students in the class. Then, the team leader introduced the method of filling out the questionnaire and provided instructions on the important aspects to be attentive about to the participants. After obtaining written informed consent from all participants, they were given a self-administered questionnaire. Once all questionnaires were responded and submitted in the class, team leaders conducted a centralized review of the questionnaires and eliminated invalid questionnaires. In the process of investigation, the investigators were instructed to properly explain to the students some items that are not easy to understand. All selected students participated in this survey voluntarily and anonymously. Before the investigation, the team leader was informed about the intelligence statuses of the students by the class teacher. We excluded 20 students who were unwilling or unwell and 9 students who had mild intellectual disability, such as autism, phenylketonuria, galactosemia, hypothyroidism, or communication difficulties owing to brain damage. We also excluded six students who had experienced childhood abuse, poor neighborhood friendliness, or bullying or had a family history of mental health disorders.

### Statistical Analysis

During data analysis, the subscales and total standardized scores were calculated for SSFD, SF-36, and SCL-90-R. The reverse entries in each subscale were forward-converted before the subscale scores for SSFD, SCL-90-R, and SF-36 were calculated. The total score was calculated as the sum of the scores in each subscale divided by the number of entries, including the disease concepts score inverted in SSFD.

Before analysis, we tested the normality of the continuous variables using the Kolmogorov–Smirnov test. Descriptive statistics are presented as $\bar{x}\pm s$ for quantitative data and *n* (%) for counts. The difference between groups was compared using the chi-square test for counts and *t*-test and one-way analysis of variance with Bonferroni *post-hoc* tests for continuous data. SF-36 subscales and the total score which represented the QoL were treated as dependent variables, whereas the demographic variables, the four subscales of SSFD, and all SCL-90-R subscales were considered independent variables in the multivariate stepwise regression analysis. The collinearity analysis was conducted before the multiple regression analysis for independent variables, and the results revealed no collinearity between independent variables (VIF < 10). Among demographic variables, only those with a significant association (*P* < 0.1) with SCL-90-R total score were selected as independent for multivariate stepwise regression.

SEM was applied to test the relations among QoL, SSFD, and SCL-90-R in senior high school students. Assuming that the three questionnaires were three latent perception constructs reflected by their respective items, a confirmatory factor analysis (34) using SEM (35) was conducted to assess the linear association among SSFD, SF-36, and SCL-90-R. The significance of indirect effects in the proposed model was evaluated by the estimates produced by SPSS 24.0 Amos (SPSS© Inc., IBM, Chicago, IL, USA). Maximum likelihood estimation was used to estimate the parameters, and several indices from a chi-square test were considered to assess the fitness of the model (36). The bootstrap method was used to test the mediating effect of mental health in systemic family dynamics and SF-36.

All statistical analyses were performed using IBM SPSS software, version 25 (SPSS© Inc., IBM, Chicago, IL, USA). *P*-values < 0.05 were considered statistically significant.

## Results

The Cronbach's α coefficient was 0.978, 0.258, and 0.123 and the split-half reliability coefficient was 0.982, 0.456, and 0.789 for QoL, SSFD, and SCL-90-R, respectively.

### Sample Characteristics

The characteristics of the respondents are summarized in Table 1. Fifty-three percent of the students (Table 1) were male, and the mean age of all students was 16.5 years. The first year (Grade 1), second year (Grade 2), and third year (Grade 3) of senior high school comprised 479 students (35.33%), 404 students (29.79%), and 473 students (34.88%), respectively. The family demographic characteristics included family income, place of residence, family composition, parents' education level, and parents' occupation. There was no difference between male and female responders in terms of family demographic characteristics, except for the occupation of their mothers. Different grades showed statistically significant differences in family monthly income, place of residence, family composition, parents' education level, and parents' occupation.

**Table 1:** Demographic characteristic of participants of different genders and with different grades [*n* (%)].

**Characteristic**	**Gender**		***P***	**Grade**			**Total**	***P***
	**Male**	**Female**		**First year**	**Second year**	**Third year**		
Income/month, Yuan			0.326					<0.001
<3,000	479 (66.16)	394 (62.34)		284 (59.29)	256 (63.37)	333 (70.4)	873 (64.38)	
3,000–5,000	172 (23.76)	164 (25.95)		123 (25.68)	104 (25.74)	109 (23.04)	336 (24.78)	
>5,000	73 (10.08)	74 (11.71)		72 (15.03)	44 (10.89)	31 (6.56)	147 (10.84)	
Place of residence			0.204					<0.001
Rural	472 (65.19)	391 (61.87)		280 (58.46)	246 (60.89)	337 (71.25)	863 (63.64)	
Urban	252 (34.81)	241 (38.13)		199 (41.54)	158 (39.11)	136 (28.75)	493 (36.36)	
Family composition			0.474					0.037
≤ 3	214 (29.56)	169 (26.74)		139 (29.02)	131 (32.42)	113 (23.89)	383 (28.25)	
4–5	414 (57.18)	371 (58.70)		278 (58.04)	212 (52.48)	295 (62.37)	785 (57.89)	
>5	96 (13.26)	92 (14.56)		62 (12.94)	61 (15.10)	65 (13.74)	188 (13.86)	
Father's education level			0.158					0.006
Below junior high school	83 (11.46)	53 (8.39)		39 (8.14)	53 (13.12)	44 (9.30)	136 (10.03)	
Junior and senior middle school	469 (64.78)	418 (66.14)		314 (65.55)	241 (59.65)	332 (70.19)	887 (65.41)	
≥College	172 (23.76)	161 (25.47)		126 (26.31)	110 (27.23)	97 (20.51)	333 (24.56)	
Mother's education level			0.104					0.023
Below junior high school	158 (21.82)	120 (18.99)		98 (20.46)	86 (21.29)	94 (19.87)	278 (20.50)	
Junior and senior middle school	451 (62.29)	386 (61.08)		284 (59.29)	237 (58.66)	316 (66.81)	837 (61.73)	
≥College	115 (15.89)	126 (19.94)		97 (20.25)	81 (20.05)	63 (13.32)	241 (17.77)	
Father' occupation			0.080					0.002
Blue collar	534 (73.76)	439 (69.46)		334 (69.73)	272 (67.33)	367 (77.59)	973 (71.76)	
White collar	190 (26.24)	193 (30.54)		145 (30.27)	132 (32.67)	106 (22.41)	383 (28.24)	
Mother' occupation			0.007					<0.001
Blue collar	592 (81.77)	479 (75.79)		359 (74.95)	310 (76.73)	402 (84.99)	1,071 (78.98)	
White collar	132 (18.23)	153 (24.21)		120 (25.05)	94 (23.27)	71 (15.01)	285 (21.02)	
Total	724	632		479	404	473	1,356	

### The Quality of Life Among Senior High School Students

The standardized scores of SF-36 are shown in Table 2. Compared with Grade 3 students, Grade 1 students had significantly higher scores for role-physical, role-emotional, bodily pain, and reported health transition (*P* < 0.05). Other subscales and the total QoL score did not significantly differ among grades. Low scores meant low QoL on all the subscales and the total score.

**Table 2:** Comparison of standardized scores of each subscale for SF-36 among different grades ($\bar{x}\pm s$).

**Subscales**	**Grades**			***P***	**Total**
	**Grade 1**	**Grade 2**	**Grade 3**		
Physical functioning	88.14, 14.42	89.31, 13.57	89.57, 13.50	0.21	88.99, 13.86
Role-emotional	70.62, 33.13^*^	67.26, 35.45	63.85, 36.62	0.018	67.02, 35.28
Role-physical	50.59, 38.01^*^	46.53, 37.52	42.42, 38.87	0.004	46.31, 35.25
Bodily pain	83.20, 17.32^*^	82.26, 17.26	80.31, 18.82	0.019	81.92, 17.80
Mental health	65.47, 16.83	66.64, 16.45	65.91, 17.00	0.798	65.83, 16.80
Vitality	63.23, 16.19	64.23, 16.49	63.37, 16.28	0.855	63.42, 16.32
Social role functioning	77.04, 19.77	76.62, 20.05	77.05, 18.07	0.945	77.00, 19.18
General health perceptions	68.44, 20.22	66.89, 20.27	67.12, 20.90	0.315	67.41, 20.50
Health transition	63.31, 23.479^*^	60.58, 26.38	58.46, 26.7	0.018	68.01, 25.57
SF-36 total	71.60, 12.33	71.54, 12.30	70.89, 12.01	0.548	71.27, 12.17

* *Compared to Grade 3, P < 0.05*.

### Systemic Family Dynamics Among Senior High School Students

The standardized scores of each subscale and the total SSFD score are shown in Table 3. Compared with Grade 3 students, Grade 2 students had lower scores for personalization and total score and higher scores for disease concepts and system logic (all, *P* < 0.05). Grade 1 students had significantly lower scores for personalization and a lower total score (*P* < 0.05). Lower scores on all four subscales and a lower total score reflect better family dynamics.

**Table 3:** Comparison of standardized scores of each subscale of SSDF among different grades ($\bar{x}\pm s$).

**Subscales**	**Grade**			***P***	**Total**
	**Grade 1**	**Grade 2**	**Grade 3**		
Family atmosphere	43.09, 9.87	44.22, 11.07	44.62, 11.04	0.071	43.96, 10.66
Personalization	45.46, 10.87^*^	44.59, 11.13^*^	47.05, 11.21	0.004	61.51, 16.07
System logic	40.38, 15.68	42.96, 17.32^*^	39.81, 15.35	0.010	40.95, 16.12
Disease concepts	60.69, 10.01	61.83, 9.95^*^	59.54, 9.71	0.003	60.63, 9.93
SSFD total	44.79, 6.27^*^	44.98, 7.16^*^	46.00, 9.64	0.014	45.27, 6.82

* *Compared to Grade 3, P < 0.05*.

### Mental Health Among Senior High School Students

The average scores of each subscale and the total SCL-90-R score are shown in Table 4. Compared with Grade 3 students, Grade 2 students had a significantly lower total score and significantly lower scores for obsessive-compulsive disorder, interpersonal sensitivity, depression, anxiety, and hostility, and Grade 1 students had a significantly lower total score and significantly lower scores for obsessive-compulsive disorder and anxiety (*P* < 0.05).

**Table 4:** Comparison of standardized scores of each subscale of SCL-90-R among different grade abbreviation expansions ($\bar{x}\pm s$).

**Subscales**	**Grade**			***P***	**Total**
	**Grade 1**	**Grade 2**	**Grade 3**		
Somatization	1.53 ± 0.58	1.57 ± 0.58	1.59 ± 0.63	0.392	1.56 ± 0.60
Obsessive-Compulsive	1.93 ± 0.66^*^	1.92 ± 0.64^*^	2.06 ± 0.71	0.002	1.97 ± 0.68
Interpersonal sensitivity	1.58 ± 0.62	1.57 ± 0.60^*^	1.66 ± 0.66	0.056	1.61 ± 0.63
Depression	1.74 ± 0.69	1.70 ± 0.65^*^	1.82 ± 0.74	0.031	1.75 ± 0.70
Anxiety	1.71 ± 0.68^*^	1.71 ± 0.64^*^	1.82 ± 0.72	0.031	1.75 ± 0.68
Hostility	1.73 ± 0.70	1.68 ± 0.69^*^	1.81 ± 0.77	0.016	1.74 ± 0.72
Phobic anxiety	1.60 ± 0.66	1.56 ± 0.62	1.65 ± 0.70	0.132	1.60 ± 0.66
Paranoid ideation	1.63 ± 0.61	1.67 ± 0.64	1.69 ± 0.65	0.377	1.66 ± 0.63
Psychoticism	1.63 ± 0.59	1.60 ± 0.58	1.67 ± 0.64	0.245	1.64 ± 0.60
SCL-90-R total	1.67 ± 0.56^*^	1.66 ± 0.56^*^	1.75 ± 0.61	0.034	1.70 ± 0.58

* *Compared to Grade 3, P < 0.05*.

### Influencing Factors of Quality of Life Among Senior High School Students

#### Comparison of Quality of Life Among Senior High School Students With Different Family Demographic Characteristics

The scores of each subscale of high school students' QoL are shown in Table 5. Compared with to females, males scored significantly higher in role-emotional and significantly lower in social role functioning (*P* < 0.05). A higher income was correlated with a higher score of physical functioning, role-physical, bodily pain, general health perceptions, and social role functioning as well as a higher total score (all *P* < 0.05). Students living in urban households had higher scores for all subscales, except VT, than those living in rural households (rural vs. urban; all *P* < 0.05). Smaller families showed a higher QoL score of physical functioning, role-physical, role-emotional, bodily pain, and general health perceptions, as well as a higher total score. Higher education level of parents was associated with higher QoL scores of physical functioning, role-physical, role-emotional, bodily pain, and general health perceptions, as well as a higher total score. Except for mental health, there were significant differences in scores of all other subscales and the total score between students with parents in blue-collar occupations vs. white-collar occupations. Students with parents engaged in white-collar occupations had higher scores in all subscales of the QoL.

**Table 5:** The scores of each subscale of quality of life and their differences in social and economic characteristics ($\bar{x}\pm s$).

**Groups**	**PF**	**RP**	**RE**	**BP**	**MH**	**VT**	**SF**	**GH**	**SF total**
**Gender**									
Male	89.58, 14.64	68.65, 35.13	48.8, 37.73^*^	82.42, 17.48	65.17, 17.2	62.86, 16.11	75.02, 0.33^*^	67.63, 20.83	71.22, 12.43
Female	88.31, 12.88	65.66, 35.16	43.93, 38.8	81.33, 18.3	66.9, 16.24	64.4, 16.5	79.1, 17.74	67.4, 20.06	71.47, 11.96
**Income/month, Yuan**									
<3,000	87.88, 14.19^*^	64.81, 35.75^*^	45.13, 37.64	80.92, 18.01^*^	65.99, 16.87	63.16, 16.21	75.97, 19.84^*^	66.06, 20.7^*^	70.51, 12.43^*^
3,000–5,000	90.65, 13.51	70.98, 34.43	49.4, 39.48	84.1, 17.42	65.8, 16.75	64.36, 16.33	78.04, 19.02	69.7, 19.56	72.65, 11.71
>5,000	91.73, 11.76	73.3, 31.87	48.3, 39.24	82.78, 17.69	66.29, 16.35	64.29, 16.83	79.97, 15.75	71.19, 20.33	73.24, 11.59
**Place of residence**									
Rural	87.18, 14.54^*^	63.79, 36.16^*^	44.77, 37.77^*^	80.34, 8.55^*^	65.27, 16.76^*^	63.05, 15.93	75.27, 19.99^*^	65.37, 20.5^*^	69.97, 12.43^*^
Urban	92.15, 11.94	73.33, 32.49	49.63, 39.05	84.67, 16.28	67.21, 16.74	64.5, 16.91	79.81, 17.58	71.28, 19.88	73.73, 11.43
**Family composition**									
≤ 3 persons	92, 12.46^*^	73.17, 32.81^*^	50.74, 39.47^*^	83.58, 17.17^*^	66.87, 17.22	64.43, 17.22	78.53, 18.34	70.32, 20.8^*^	73.27, 12.1^*^
4–5 persons	88.11, 14.05	65.99, 35.32	44.71, 37.66	81.63, 17.94	65.57, 16.53	63.13, 15.85	76.5, 19.66	66.96, 20	70.79, 12.12
>5 persons	86.52, 14.76	60.51, 37.46	45.57, 38.05	79.69, 18.74	65.81, 16.91	63.72, 16.3	75.35, 19.37	64.14, 21.12	69.67, 12.39
**Father's education level**									
Below junior high school	85.99, 14.88^*^	61.58, 37.63^*^	44.61, 37.23	78.4, 18.19^*^	63.18, 16.72	61.65, 16.16	75.16, 20.34	63.85, 19.51^*^	68.57, 12.49^*^
Junior and senior middle school	88.42, 14.15	66.35, 34.78	45.36, 37.99	81.53, 18.15	66, 16.92	63.34, 16.28	76.75, 18.82	66.92, 20.71	70.96, 12.35
≥College	91.71, 12.15	72, 34.69	50.45, 39.37	84.35, 16.67	67.05, 16.31	65, 16.36	78.08, 19.97	70.61, 19.85	73.47, 11.38
**Mother's education level**									
Below junior high school	85.34, 15.27^*^	60.34, 36.03^*^	39.09, 35.25^*^	79.3, 18.37^*^	63.31, 17.32^*^	61.44, 17.15^*^	74.66, 20.23^*^	62.21, 21.1^*^	67.93, 12.87^*^
Junior and senior middle school	89.15, 13.8	67.65, 34.97	46.75, 38.29	81.76, 17.91	66.28, 16.66	63.64, 15.73	76.89, 19.22	68.28, 19.8	71.59, 11.89
≥College	92.61, 11.1	73.86, 33.48	54.36, 40.15	85.44, 16.61	67.97, 16.23	65.81, 17.02	79.62, 17.99	70.99, 20.97	74.39, 11.61
**Father's occupation**									
Blue collar	88.06, 14.2^*^	65.34, 35.76^*^	44.91, 7.78^*^	81.07, 18.05^*^	65.44, 16.74	62.66, 6.24^*^	76.14, 19.4^*^	66.07, 20.4^*^	70.38, 12.39^*^
White collar	91.34, 12.65	72.13, 33.13	50.65, 39.32	84.03, 17.24	67.32, 16.82	65.9, 16.25	78.88, 18.81	71.2, 20.2	73.76, 11.4
**Mother' occupation**									
Blue collar	88.1, 14.23^*^	65.97, 5.43^*^	44.88, 37.61^*^	81.17, 8.01^*^	65.6, 16.68	62.96, 16.17^*^	76.06, 19.64^*^	66.54, 20.18^*^	70.6, 12.28^*^
White collar	92.3, 11.8	72.11, 33.74	52.75, 0.21	84.71, 17.1	67.38, 17.09	65.88, 16.64	80.16, 17.44	71.18, 21.17	74.1, 11.55

* *P < 0.05*.

*PF, physical functioning; RP, role-physical; RE, role-emotional; BP, bodily pain; MH, mental health; VT, vitality; SF, social role functioning; GH, general health perceptions*.

#### Multiple Linear Regression Analysis on Influencing Factors of Quality of Life

The results of multiple linear regression analysis are shown in Table 6. The model included demographic variables (place of residence, family income, family composition, father's/mother's education level, and father's/mother's occupation), the four sub-subscales of SSFD, and the eight subscales of SCL-90-R. All the included factors could account for 37.5% of the variance of the total SF-36 (R^2^ = 0.375, *F* = 29.592, *P* < 0.001).

**Table 6:** Multiple linear regression analysis of demographic, SSFD, and SCL-90-R parameters by SF-36 subscales^a^.

**Factors**	**PF**	**RP**	**RE**	**BP**	**MH**	**VT**	**SF**	**GH**	**SF-36 total**
*R^2^*	0.138	0.098	0.158	0.187	0.248	0.258	0.072	0.239	0.364
*F-*values*/P-*values	26.976/ <0.001	24.649/ <0.001	42.344/ <0.001	77.792/ <0.001	88.876/ <0.001	67.085/ <0.001	14.856/ <0.001	53.052/ <0.001	29.592/ <0.001
**Grade**									
Grade 3^b^	—	−0.055^*^	—	—	—	—	0.070^**^	—	—
Place of Residence	−0.131^**^	−0.068^*^	—	−0.074^**^	—	—	—	−0.102^**^	−0.085^**^
Mother's profession	—	—	0.072^**^	—	—	—	—	—	—
Father's profession	—	—	—	—	—	0.050^*^	—	—	—
**Mother's education**									
Junior and senior middle school^c^	—	—	—	—	—	—	—	0.062^*^	—
Family atmosphere	0.069^**^	—	—	—	0.112^**^	−0.122^**^	—	−0.148^**^	−0.088^**^
Personalization	—	—	−0.055^*^	—			—	—	—
System logic	−0.143	—	—	—	−0.165^*^	−0.088^**^	−0.139^**^	−0.177^**^	−0.180^**^
Disease concepts	—	−0.108^**^	—	—	−0.059^*^	−0.070^**^	−0.072^*^	—	−0.017^**^
Somatization	−0.148^**^	−0.143^**^	0.131^**^	−0.322^**^	−0.080^*^		−0.150^**^	−0.170^**^	−0.170 ^**^
Obsessive-Compulsive	0.160^**^	−0.113^**^	−0.218^**^	—	—	−0.171^**^		—	—
Interpersonal sensitivity	0.128^*^	—	—	0.094^*^	—		0.152^**^	0.112^*^	—
Depression	−0.186^**^	—	−0.215^**^	−0.202^**^	−0.362^**^	−0.358^**^	—	−0.220^**^	−0.372^**^
Anxiety	—	—	—	—	—	—	—	—	—
Hostility	—	—	−0.078^*^	—	—	—	0.121^**^	—	—
Phobic anxiety	−0.179^**^	—	—	—	—	0.072^*^	−0.156^**^	—	—
Psychoticism	—	—	—	—	—	—		−0.139^**^	—

a *The data in the table are the standardized partial regression coefficients in the results of multiple regression analysis, and “—” indicates that this factor is not included in the model*.

b *Reference as grade 1*.

c *Reference as below junior high school*.

** *P < 0.01*,

* *P < 0.05*.

*PF, physical functioning; RP, role-physical; RE, role-emotional; BP, bodily pain; MH, mental health; VT, vitality; SF, social role functioning; GH, general health perceptions*.

Only the variable of place of residence was entered in the model for the demographic variables. The somatization and depression subscales of SCL-90-R were significantly negatively associated with the SF-36 total scores (standardized partial regression coefficients β = −0.170, β = −0.372, respectively). Albeit moderately, disease concepts, family atmosphere, and systematic logic were negatively associated with the SF total scores (β = −0.071, β = −0.088, and β = −0.180, respectively).

#### Correlations Among SF-36, SCL-90-R, and SSFD

According to the multiple stepwise regressions, the variance contribution of family dynamics to the QoL was relatively small but that of mental health was relatively large. Therefore, family dynamics may have indirect effects on the QoL of students through mental health problems.

The items in the questionnaire were statistically significant (*P* < 0.01) in the resulting SEM, of which the chi-square test was statistically significant (χ^2^ = 1425.85, *P* < 0.0001), the comparative fit index was 0.930 (>0.90), the root-mean-square error of approximation was 0.071 (<0.1), the goodness-of-fit index was 0.902 (>0.9), and the normed fit index was 0.921 (>0.9).

**Figure 1:**
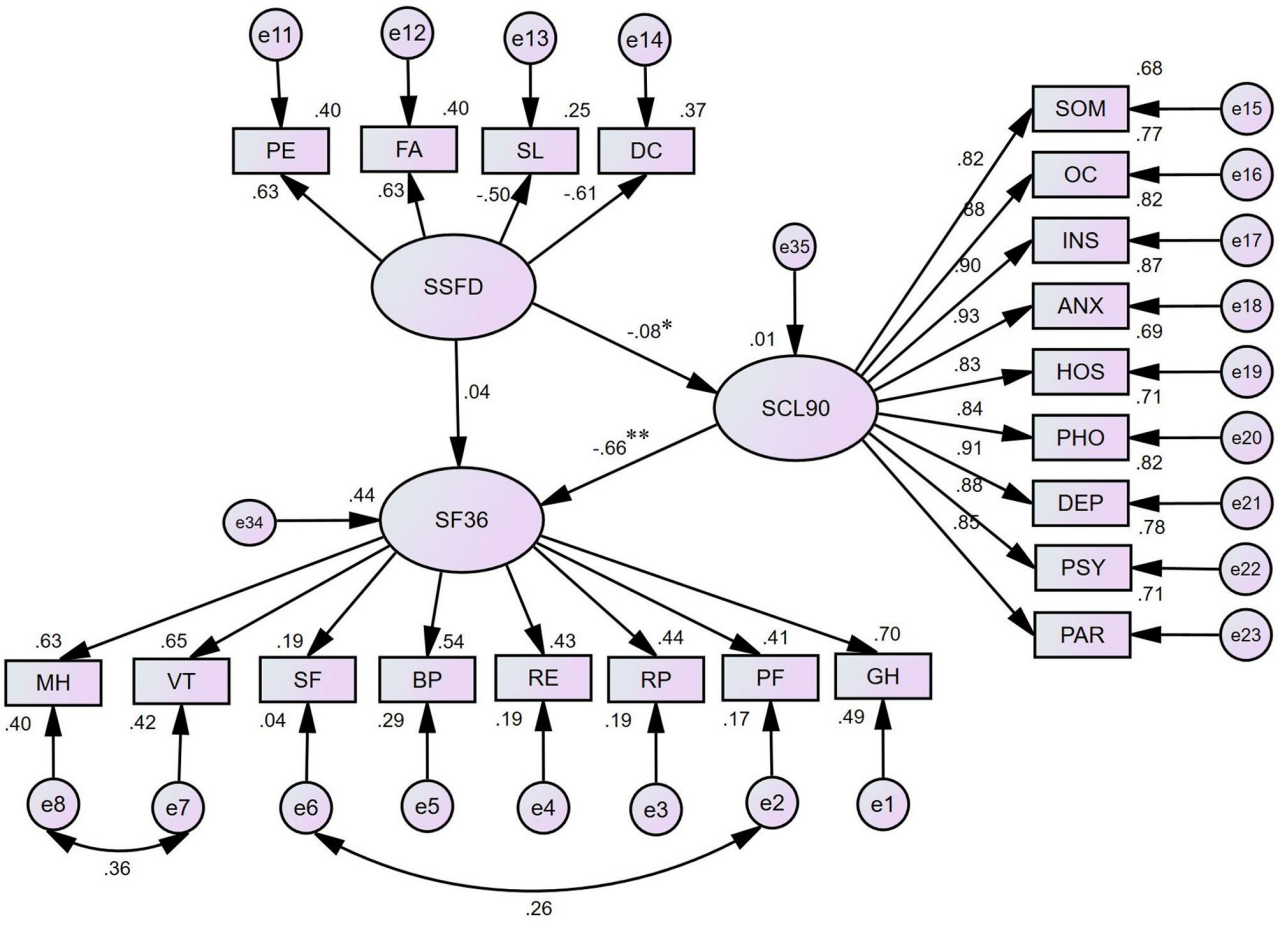
The structure equation modeling for SF-36, SSFD and SCL-90-R. ^*^P < 0.05; ^**^P < 0.01; SF 36, the 36-item short form health survey; SSFD, the self-rating scale of systemic family dynamics; SCL-90-R, the symptom checklist-90-revised; MH, mental health; VT, vitality; SF, social role functioning; BP, bodily pain; RE, role-emotional; RP, role-physical; PF, physical functioning; GH, general health perceptions; SOM, somatization; OC, obsessivecompulsive; INS, interpersonal sensitivity; ANX, anxiety; HOS, hostility; PHO, phobic anxiety; DEP, depression; PSY, psychoticism; PAR, paranoid ideation; PE, personalization; FA, family atmosphere; SL, system logic; DC, disease concepts.

The standardized parameter estimates are shown in Figure 1. As indicated, SF-36 score was not significantly correlated with the SSFD but had a strong negative correlation with SCL-90-R (standardized regression coefficient β = −0.04, *p* = 0.178; β = −0.66, *p* < 0.001). Furthermore, the SSFD score showed a moderately negative correlation with the SCL-90-R score (β = −0.08, *p* = 0.018). Therefore, the mediating effects were analyzed, with the SCL-90-R score as the intermediate outcome between the SSFD score as the exogenous variable and the SF-36 score as the final outcome. The result of SEM shows that the SSFD score fully mediated the association between the SCL-90-R score and SF-36 score (indirect effect coefficient 0.055; 95% CI, 0.012–0.106; *P* = 0.042). For the explanatory power of SEM, the family dynamics and mental health accounted for 57% of the variance in the mental health value.

## Discussion

The score of role-emotional problems was 46.31 ± 35.25, and the standard deviation was large, which indicates that the QoL of high school students needs to be improved, particularly in the context of role-emotional problems, and that this aspect warrants attention of parents and schools alike. High school students are under a lot of achievement pressure, which can easily lead to mental tension, anxiety, and interpersonal problems, thus indirectly affecting their QoL (37, 38). Therefore, special attention is warranted to meet the emotional needs of high school students to improve their QoL. The analysis of the scores of each subscale of the QoL in different grades shows that the scores of Grade 3 students are significantly lower than those of Grades 1 and Grade 2 students in the three subscales of physical functioning, role-emotional, and bodily pain, which reflects the poor QoL caused by heavy schoolwork because of the immense pressure to graduate from high school (8).

The multivariate linear regression model showed that in high school students, the place of residence had a positive impact on the QoL, with students living in the city having a better QoL than those living in rural areas; this aspect may be influenced by the family's economical condition and support abilities because urban students' living conditions are typically better than those of countryside students. The QoL of senior high school students was evidently affected by systemic family dynamics and mental health. Among the subscales of systemic family dynamics, family atmosphere showed the highest correlation with the QoL, followed by systematic logic and disease concepts. Higher scores of this subscale were associated with a more relaxed, harmonious, and happy family atmosphere and vice versa. This is in agreement with the study conducted by ZENG Wei-nan who reported that family support can affect the QoL (39). Adolescents with good disease concepts have scientific understanding and an optimistic attitude toward diseases; therefore, they can dialectically view the relationship between disease and health. This approach is of great benefit for health maintenance and recovery.

The multivariate linear regression model also reports that somatization and depression variables of mental health were crucial risk factors for a lower QoL of senior high school students. Somatization is among the most common mental health problems exhibited by adolescents. It involves the presentation of physical symptoms that are either disproportionate or inconsistent with history, physical examination, laboratory, and other investigative findings. It mostly manifests as headache, chest pain, vomiting, and fatigue. In pediatric outpatient clinics, 2–24% of patients present with functionalized somatic symptoms in the United States (40). The total detection rate of functional somatization symptoms in Chinese adolescents is 7.6% (41). Depression has a wide array of symptoms affecting somatic, cognitive, affective, and social processes, which is a common problem for adolescents. Therefore, it is feasible to improve the QoL by changing somatization and depressive symptoms (42).

The results of SEM show that the SSFD score fully mediated the association between the SCL-90-R score and SF-36 score. Therefore, it is possible to improve the QoL of high school students by improving and enhancing system family dynamics, and more attention should be paid to discovering and solving their mental health problems. Teenagers who live in a good family atmosphere have more positive emotions, have a sense of security, and are more positive and optimistic. In this way, the psychological development of high school students can be healthy, which promotes healthy growth of the body and a better QoL in young people.

With the development of the social economy and the improvement in people's living standards, some demographic variables related to the QoL of high school students may have changed in recent years, such as higher household income. The COVID-19 pandemic may have worsened the existing mental health problems in adolescents and increased the risk of future mental health issues (43). Therefore, the QoL of senior high school students and the family and mental health factors affecting the QoL may have changed, and future research is needed to confirm the relationship.

### Limitations

This study has some limitations. This was a cross-sectional study and true causality cannot be inferred. To show that systemic family dynamics and mental health influence the QoL, it would be necessary to measure systemic family dynamics and mental health at one time point and later on measure the QoL at another time point after addressing the concerns. In addition, many other factors besides basic sociodemographic variables, systemic family dynamics, and mental health can affect the QoL of adolescents. For example, having a physical illness would greatly affect the QoL of an adolescent; however, given the cross-sectional design, these aspects could not be considered in this study. Moreover, more recent data that would reflect the QoL situation in recent years were not available. We intend to do a survey to further explore the other influencing factors of QoL on senior high school students, confirm the relationships between these factors and students' QoL, and understand the changes in QoL. Finally, unfortunately, no family medical history was collected during the investigation. Students having good disease concepts may be attributable to a family member suffering from a certain disease; however, this requires further investigation to draw conclusions.

## Conclusion

It was found that the QoL of senior high school students was related to their gender, family income, place of residence, family composition, and parents' educational level and occupation types. Mental health fully mediated the association between systemic family dynamics and QoL in senior high school students. It is possible to improve the QoL of high school students by improving systemic family dynamics and discovering and addressing their mental health problems.

## Data Availability Statement

The datasets used and analysed during the current study are available from the corresponding author on reasonable request.

## Ethics Statement

Ethical review and approval were not required for the study on human participants in accordance with the local legislation and institutional requirements. Written informed consent was obtained from all adolescents as well as from their parents/guardians.

## Author Contributions

LS was responsible for the study conception and design. ZY was responsible for manuscript preparation. YS, YL, and HZ contributed to data acquisition, analysis, and interpretation. YY and YW contributed to the review of the data. LS and YZ contributed to the critical revision of the manuscript, obtained funding, and supervised the research. All author(s) have read and approved the final manuscript.

## Funding

This research was supported by National Natural Science Foundation of China (81773540) and Key R&D Program of Shaanxi Province (2021SF-193).

## Conflict of Interest

The authors declare that the research was conducted in the absence of any commercial or financial relationships that could be construed as a potential conflict of interest.

## Publisher's Note

All claims expressed in this article are solely those of the authors and do not necessarily represent those of their affiliated organizations, or those of the publisher, the editors and the reviewers. Any product that may be evaluated in this article, or claim that may be made by its manufacturer, is not guaranteed or endorsed by the publisher.

## Abbreviations

QoL: quality of life
SF-36: the 36-item Short Form Health Survey
SSFD: the Self-rating Scale of Systemic Family Dynamics
SCL-90-R: the Symptom Checklist-90-Revised
PF: Physical functioning
RP: Role-physical
BP: Bodily pain
GH: General health perceptions
VT: Vitality
SF: Social role functioning
RE: Role-emotional
MH: Mental health.
